# Insights into remediation effects and bacterial diversity of different remediation measures in rare earth mine soil with SO_4_^2−^ and heavy metals

**DOI:** 10.3389/fmicb.2023.1050635

**Published:** 2023-03-23

**Authors:** Xiao Yan, Bowen Gao, Jianlei Wang, Xuezhe Zhu, Mingjiang Zhang

**Affiliations:** ^1^GRINM Resources and Environment Tech. Co., Ltd., Beijing, China; ^2^National Engineering Research Center for Environment-Friendly Metallurgy in Producing Premium Non-Ferrous Metals, GRINM Group Co., Ltd., Beijing, China; ^3^School of Metallurgy, Northeastern University, Shenyang, China; ^4^GRIMAT Engineering Institute Co., Ltd., Beijing, China

**Keywords:** sulfate-reducing bacteria (SRB), SO_4_^2−^, heavy metals, rare earth mines soil, soil bacterial community, bacterial function

## Abstract

The increased demand for rare earth resources has led to an increase in the development of rare earth mines (REMs). However, the production of high-concentration leaching agents (SO_4_^2−^) and heavy metals as a result of rare earth mining has increased, necessitating the removal of contaminants. Here, a series of experiments with different remediation measures, including control (CK), sulfate-reducing bacteria (SRB) alone (M), chemicals (Ca(OH)_2_, 1.5 g/kg) plus SRB (CM-L), chemicals (Ca(OH)_2_, 3.0 g/kg) plus SRB (CM-M), and chemicals (Ca(OH)_2_, 4.5 g/kg) plus SRB (CM-H), were conducted to investigate the removal effect of SO_4_^2−^, Pb, Zn, and Mn from the REM soil. Then, a high-throughput sequencing technology was applied to explore the response of bacterial community diversity and functions with different remediation measures. The results indicated that CM-M treatment had a more efficient removal effect for SO_4_^2−^, Pb, Zn, and Mn than the others, up to 94.6, 88.3, 98.7, and 91%, respectively. Soil bacterial abundance and diversity were significantly affected by treatments with the inoculation of SRB in comparison with CK. The relative abundance of *Desulfobacterota* with the ability to transform SO_4_^2−^ into S^2−^ increased significantly in all treatments, except for CK. There was a strong correlation between environmental factors (pH, Eh, SO_4_^2−^, Pb, and Zn) and bacterial community structure. Furthermore, functional prediction analysis revealed that the SRB inoculation treatments significantly increased the abundance of sulfate respiration, sulfite respiration, and nitrogen fixation, while decreasing the abundance of manganese oxidation, dark hydrogen oxidation, and denitrification. This provides good evidence for us to understand the difference in removal efficiency, bacterial community structure, and function by different remediation measures that help select a more efficient and sustainable method to remediate contaminants in the REM soil.

## Highlights

- The SRB system has a positive effect on SO42- removal and heavy metals stabilization.
- The treatments with the inoculation of SRB clearly increased functional microbial community abundance and affected microbial community structure.
- Desulfobacterota was most sensitive to SO42- and heavy metals.
- Functional microbes (Desulfosporosinus, Desulfitobacterium, Desulfobulbus and Dethiosulfovibrio) promotes sulfur cycling and provides sustainable ecological remediation in the REEs mine soil.

## Introduction

Rare earth elements are recognized as critical raw materials that are an important part of all high-tech devices, including electronics (TV, autocatalytic converters, and telephone), superconductors (high energy particle accelerator, maglev train, and energy storage devices), and fluorescent materials (indicator, glass additives, and clothes) (Lima and Ottosen, 2021). A series of environmental problems result from the development and utilization of rare earth mining (Amol et al., 2022). According to statistics, about 3,000 mg/l of SO_4_^2−^ is produced in the rare earth industry every year (Zhou et al., 2022) in addition, some heavy metals (Pb, Zn, and Cu) from surrounding mines may be migrated to the REM soil (Du et al., 2022). The combined pollution of SO_4_^2−^ and heavy metals not only destroys the soil quality and decreases the crop yield but also harms human health through the food chain (Sharma et al., 2021). More importantly, the contaminants could spread with rainfall or water flow, which results in the expansion of the polluted area (Tran et al., 2021; Wang F. et al., 2022). Considering potential risks, the remediation of these combined pollutants in rare earth soil has been widely concerned (Li L. et al., 2022). At present, the main remediation technologies including physical, chemical, and biological methods are applied to treat SO_4_^2−^ and heavy metals of rare earth soil (Ali et al., 2022). Chemical agents, such as Ca(OH)_2_, possess heavy metal absorption capability to remove dissolved heavy metals (Pb and Zn) effectively at high concentrations of up to 1,000 mg/L (Du et al., 2022). In addition, Ca(OH)_2_ is often used as a reagent to regulate the pH of contaminated soil rapidly. However, excessive application of chemicals has the disadvantages of high operational cost, damaging soil quality, and production of secondary pollutants (Mokoena et al., 2022). In comparison with physical and chemical methods, the biological method was anticipated as one of the most promising technologies due to low-cost, environment-friendly, and sustainable remediation (Saxena et al., 2021). The combined pollution of SO_4_^2−^ and heavy metals was transformed and immobilized by functional microbes through mechanisms of bioreduction, biosorption, and biomineralization (Zhang et al., 2020).

Sulfate-reducing bacteria (SRB) are a typical functional bacterial species that can transform SO_4_^2−^ to S^2−^ by acting as the terminal electron acceptor in the process of dissimilatory sulfate reduction, which is an important reaction in global sulfur cycling (Santos et al., 2022). Previous studies indicated that the SRB belongs to different genera including *Desulfomicrobium*, *Desulfitobacterium*, *Desulfobulbus*, and *Dethiosulfovibrio* (Yang Z. et al., 2021; Tang et al., 2022). It has been reported that SRB are widely distributed in anaerobic environments, such as sewage sludge, polluted oil field, mine tailings, animal intestines, and even acidic mine drainage (Zhu et al., 2020; Gu et al., 2021). SRB can use SO_4_^2−^ as electron acceptors to reduce SO_4_^2−^ to S^2−^. Then, S^2−^ can remove heavy metals in the water solution by synthesizing a variety of insoluble sulfides precipitation including PbS, ZnS, CuS, and MnS (Lv et al., 2022a,b). Previous studies concluded that SRB can effectively remove heavy metals (Pb, Zn, Cu, Cd, Cr, and U) in the water solution from acid mine drainage and sustainably improve the ecological environment (Gu et al., 2021; Wang et al., 2021; You et al., 2021; Yang et al., 2021a). For example, Yang X. et al. (2021) and Yang Z. et al. (2021) inoculated SRB into a biological filter disk carrier, which can decline SO_4_^2−^ concentration from wastewater systems by over 90% in 60 days (Yang Z. et al., 2021). Lv et al. (2022a,b) reported that the 99% of U(VI) was solidified by SRB when the initial concentration of U(VI) is about 5 mg/L (Li J. et al., 2022). Therefore, the SRB was widely used to treat wastewater containing SO_4_^2−^ and heavy metal contamination of soil (Li J. et al., 2022). However, for an extremely high concentration of SO_4_^2−^ combined with heavy metal contamination soil, signal bacterial technology (SRB) is difficult to achieve the anticipated effects in the short term (Ali et al., 2022). More importantly, several contaminated sites containing SO_4_^2−^ were considered too acidic for the colonization of SRB. Therefore, the combination methods of chemical and biological are considered the most efficient technology in harsh environments (Zhao et al., 2019).

The leaching reagents were widely used in the process of rare earth element leaching (Traore et al., 2022). So numerous studies focused on the screening and application of leaching reagents in the process of rare earth element leaching from the REM (Talan and Huang, 2022; Zhou et al., 2022), but a large amount of leaching reagents not only affected soil quality but also increased ecological risk for rare earth ores after closure (Mokoena et al., 2022). However, few studies focused on treating leaching reagents and variation of microbial community succession in the REM. Recent studies have found that combined pollution of SO_4_^2−^ and heavy metals strongly affected soil quality and further regulated bacterial community structure and functions in process of treating acid mine drainage (Villegas-Plazas et al., 2019). Similarly, it was possible that bacterial community structure and ecological functions in the REM soil were significantly affected by combined pollution of SO_4_^2−^ and heavy metals. It may be a close association and intense interaction between contamination removal efficiency and bacterial dominant flora. In order to find the truth from above conjecture, the main objectives of this study were as follows: (1) to compare the removal effects of SO_4_^2−^ and heavy metals from the REM soil under different remediation measures; (2) to explore the differences of bacterial diversity, community structure, and analyze the effects between contaminants and bacterial community structure in the REM soil experiments under different remediation measures; and (3) to predict the abundance differences of metabolism or other ecological-related functions (such as nitrification and denitrification) under different treatments.

## Materials and methods

### Sampling and analysis

The contaminated soil used in this study was collected from the closure of the REM, located in Ganzhou, Jiangxi Province, China (25°42′N, 115°07′E). A total of 12 points were selected in the REM, and all samples were taken with a shovel at a spatial interval of 20 m for each sample from the surface of approximately 0–20 cm. Then, the samples were stored in polyethylene seal pockets at 4°C and transported to the laboratory within 24 h for further measurement of the initial concentration of target pollutants. All samples were mixed over 20 times to form a sample for the remediation experimental study of columns. The chemical composition of the REM soil sample was described in our previous study, which primarily contains high concentrations of SO_4_^2−^ and heavy metals such as Pb, Zn, and Mn (Zhou et al., 2022). The SO_4_^2−^ concentration was determined by referring to the turbidimetric method (Tayar et al., 2022). According to the methods described by Singh, the concentration of heavy metals including Pb, Zn, and Mn from the leaching solution was determined by ICP-MS (Agilent Technologies 7700x, United States) (Singh et al., 2022). In addition, the pH and Eh were measured by a portable multi-parameter digital analyzer (HQ40d, HACH, United States).

### Bacteria and rejuvenation

The SRB were isolated from activated sludge (Yan et al., 2019) and preserved in the National Engineering Laboratory of Biohydrometallurgy, China. First, 10 mL of the SRB bacterial fluid was added to 90 mL of LB medium (sterilized at 121°C for 30 min) to prepare an expand culture at 30°C for 48 h. Then, the supernatant liquid was transferred into the fresh LB medium with an inoculation volume of 10%. When the OD_600_ (the optical density value was measured when the wavelength is set at 600 nm) in the culture system was reaching to 0.4, the supernatant liquid was transferred again for rejuvenation. Finally, the activated SRB solution was added to remediate systems with 10% inoculation volume.

### Experimental design

First, 10 cm quartz sand was placed at the bottom of the PVC experimental columns (0.7 cm diameter, 50 cm height). Then, a 1.4 kg soil sample after well mixing was added to each column. This experiment comprised five treatments, including no chemicals and SRB treatment (CK); inoculation with SRB alone (M); adding Ca(OH)_2_ at a dosage of 1.5 g/kg for 7 days in the soil sample for treatment priority, and then, inoculated SRB for sustainable treatment for contaminants (CM-L); adding Ca(OH)_2_ at a dosage of 3.0 g/kg for 7 days in the soil sample for treatment priority, and then, inoculated SRB for sustainable treatment for contaminants (CM-M); adding Ca(OH)_2_ at a dosage of 4.5 g/kg for 7 days in the soil sample for treatment priority, and then, inoculated SRB for sustainable treatment for contaminants (CM-H). For each treatment (CK, M, CM-L, CM-M, and CM-H), three replicates were set to reduce experimental error. A total of 15 columns were used in this experiment. The treatment time was 42 days, and the leaching solution of each column was collected every 7 days for the detection of the variation of pH, Eh, SO_4_^2−^ and heavy metal (Pb, Zn, and Mn) concentrations in different remediation measure systems. When the significant remediation effect was achieved (about 30 days), the bacterial community abundance, diversity, and functions of the REM soil under different treatments were measured by high-throughput sequencing technology.

### DNA extraction

DNA was extracted from the REM soil under different treatments using a DNeasy PowerSoil Kit (QIAGEN, Germany) according to the manufacturer’s instructions. The V3–V4 region of the 16S rRNA gene was amplified by polymerase chain reaction (PCR) with primers 338F (5′-ACTCCTACGGGAGGCAGCAG-3′) and 806R (5′-GGACTACHVGGGTWTCTAAT-3′) (Lv et al., 2022a,b). Each sample was amplified with three technical replicates under the following conditions: 94°C for 5 min, and then 30 cycles of 94°C for 30 s, 56°C for 30 s, and 72°C for 45 s, and a final extension at 72°C for 7 min. PCR products were purified using a QIAamp 96 PowerFecal QIAcube HT kit (QIAGEN, Germany). The purified products were mixed at equimolar concentrations and then applied to sequence on an Illumina HiSeq 2500 (PE250) platform at Shanghai Major Biomedical Technology Co., Ltd.

### Bioinformatics analysis

The raw sequences were processed using the QIIME pipeline (Ma et al., 2022). We used the denoising software DADA2 to remove low-quality reads, putative chimera, and then, the result was parsed into amplicon sequence variants (ASVs) with default quality settings (Callahan et al., 2017). Based on the Silva V138 (99%) reference database, the ASVs were used as the more particular taxonomy unit than the species (Yang et al., 2022). The detailed taxonomic affiliation of ASVs was obtained from the National Center for Biotechnology Information (NCBI) website in order to conduct further analysis. ASVs present in only one sample was removed from the final dataset because they could be remaining sequencing errors not detected by DADA2, resulting in 1764 ASVs and 946,312 reads in the final dataset. The sequence counts per sample were rarefied to the smallest individual sample sequence.

### Statistical analysis

Microsoft Excel and Origin 2017 software were used for the statistical analysis of the variation of physical and chemical properties and contaminations in the experiment system. The diversity metrics (species’ richness and Shannon diversity) and bacterial community composition on the phylum/genus level (Bray–Curtis dissimilarity matrices) were analyzed on the online tool of Majorbio Cloud Platform.^1^ The canonical correlation analysis (CCA) and Spearman correlational analyses were performed to examine the relationship between the contaminations and bacterial community structure. FAPROTAX was used to predict the biochemical cycle of different treatments. The prokaryotic taxa were mapped to metabolic or other ecologically relevant functions using FAPROTAX software based on the literature on cultured representatives (Ma et al., 2022).

## Results and discussion

### Effect of different remediation measures on pH, Eh, SO_4_^2−^, and heavy metals in the REM soil

**Figure 1:**
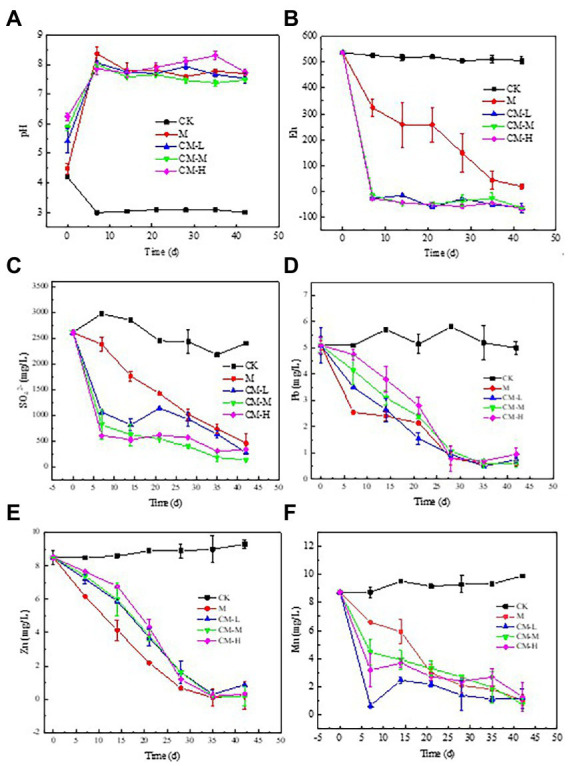
Variation of physicochemical properties [**(A)** pH, **(B)** Eh] and the contaminants [**(C)** SO_4_^2−^, **(D)** Pb, **(E)** Zn, and **(F)** Mn] in the experimental system of REM soil by different remediation measures: CK (no amendment), M [sulfate-reducing bacteria (SRB) alone], CM-L [chemicals (Ca(OH)_2_, 1.5 g/kg) plus SRB], CM-M [chemicals (Ca(OH)_2_, 3.0 g/kg) plus SRB], and CM-H [chemicals (Ca(OH)_2_, 4.5 g/kg) plus SRB].

In order to understand the remediation effects of different measures on removing contaminants, the variation of pH, Eh, SO_4_^2−^, and heavy metals concentration was monitored in this study. The results showed that the initial values of soil pH and Eh were 4.20 and 537 mV, respectively, which means that this site was still in a strongly acidic and extremely oxidized state. Compared with CK, an increasing trend of pH is presented in the others. After 7 days, the pH reached 7.5 and continued throughout the treatment process (Figure 1A). With increasing pH, there was a decrease in Eh value in each treatment, but the decrease varied between treatments. The variation of Eh value in the CM-L, CM-M, and CM-H was rapid, decreasing to 0 mV at 7 days and continuing (Figure 1B), but a relatively slight decrease from 537 mV to 19 mV was presented in the M. The changes in Eh showed that the strong oxidation state was gradually transformed into the reduction state.

The phenomenon of increased pH with decreasing Eh may be influenced by two aspects: neutralization of chemical agents and transformation of microorganisms. The addition of Ca(OH)_2_ directly neutralizes H^+^ and rapidly changes pH and Eh values. This result was consistent with Florentino’s conclusion that alkaline chemicals could quickly neutralize the acid from acid mine wastewater and affect redox conditions (Florentino et al., 2015). The SRB was a kind of common functional microbe that changed the system’s extreme oxidization to a reduction state by converting SO_4_^2−^ to S^2−^, which was a process of acid consumption and alkali production (Villegas-Plazas et al., 2019).

In this study, we investigated the effects of different remediation measures on the SO_4_^2−^ removal effects. As shown in Figure 1C, the SO_4_^2−^ concentration in the CK was first decreased and then kept constant (about 2,700 mg/L). The increased SO_4_^2−^ concentration in 7 days may be due to the dissolution of sulfate compounds in the soil under strongly acidic conditions. However, SO_4_^2−^ concentration in treatments, except the CK, decreased over time. Among them, when the Ca(OH)_2_ concentration was increased from 1.5 to 4.5 g/L, the variation of SO_4_^2−^ concentration followed an order of the CM-M (from 2,610 to 138.5 mg/L) > the CM-L (from 2,610 to 278.9 mg/L) > the CM-H (from 2,610 to 339.6 mg/L). Such an anticipated removal effect of SO_4_^2−^ by the addition of the middle Ca(OH)_2_ concentration was reported in previous studies (Liu et al., 2022; Yang et al., 2022).

The SO_4_^2−^ concentration in the CM-L was relatively higher than that of the CM-M, which may have a relationship with the relatively lower Ca(OH)_2_ concentration. Meantime, it takes longer to convert SO_4_^2−^ to H_2_S by the SRB (Santos et al., 2022). In addition, CaSO_4_ as a low solubility substance released SO_4_^2−^ with time at relatively low pH conditions (Xing et al., 2022). In comparison with the CM-L, CM-M, and CM-H, the variation of SO_4_^2−^ concentration in the M was relatively slow, from 2,610 mg/L to 460.5 mg/L during the remediation process, which was explained that the SRB needs to be satisfied for more time to adapt to a new environment first, and then, the population and activity of SRB gradually increase over time (Tang et al., 2022). This result was consistent with of the variation of Eh values. With the increase of SRB abundance, the systems gradually transformed from the oxidized state to the reduced state, and then, the SO_4_^2−^ transformed into H_2_S by RSB under the reduction conditions (Li W. et al., 2022). After remediation, SO_4_^2−^ concentrations in the earlier treatment systems were all below the effluent discharge standard (800 mg/L).

The studied soil was collected from the REM under closed storage conditions in the south of Ganzhou city, Jiangxi Province, China. According to the previous investigation, many heavy metal mines such as lead–zinc gather around this studied site (Li J. et al., 2022). Due to the lack of appropriate management, the concentrations of Pb and Zn in the surrounding soil exceeded the standard to varying degrees (Ali et al., 2022). In this study, the Pb, Zn, and Mn concentrations were analyzed. The results showed that Pb, Zn, and Mn concentrations in the different remediation measures, except the CK, presented a declining trend over time (Figures 1D–F). In comparison with the CM-L, CM-M, and CM-H, the decline of Pb, Zn, and Mn concentrations in the M was relatively significant, ranging from 5.15, 8.56, 8.49 mg/L to 0.51, 0.22, and 1.05 mg/L, respectively. The removal rate reached 90.1, 97.4, and 87.6%. Such a predictable removal effect was mainly attributed to the large number and high activity of SRB (Yang et al., 2022). It was clear that abundant SRB in the system could increase the chance of contacting SO_4_^2−^, and the higher activity of SRB in the system was a key to transforming SO_4_^2−^ rapidly (Wang C. et al., 2022). When the population and activity of SRB are met at the same time, SRB can quickly and efficiently convert from SO_4_^2−^ to H_2_S, and then, the H_2_S combined with heavy metal ions (Pb and Zn) to form stable compound precipitation (PbS and ZnS), so as to achieve the purpose of solidification of heavy metals in the REM soil (Lv et al., 2022a,b). For the collaborative remediation group with the addition of Ca(OH)_2_ and SRB, the remediation effects of Pb, Zn, and Mn were as follows: the CM-M (from 5.15, 8.56, and 8.49 to 0.60, 0.17, and 0.76) > the CM-L (from 5.15, 8.56, and 8.49 to 0.75, 0.87, and 1.19) > the CM-H (from 5.15, 8.56, and 8.49 to 0.95, 0.32, and 1.30). Such a CM-M treatment was anticipated as in the previous results. The addition of an appropriate amount of Ca(OH)_2_ was beneficial to the removal of contaminants due to suitable soil pH. Meanwhile, the growth of the SRB would be inhibited in more acid or basic conditions. In comparison to CK and M, the rapid change in heavy metal concentration over 7 days may be attributed to the high efficiency of Ca(OH)_2_. Subsequently, suitable pH and moderate heavy metal concentration provided a favorable condition for the removal of contaminants by SRB. This was a measure to realize the sustainable remediation of the REM soil under closed storage conditions. Previous study results showed that the SRB had strong tolerance against heavy metals and had a special capacity for solidified heavy metals, such as Pb, Zn Cu, U, Cr, and Mn (Lin et al., 2022). Solidification of heavy metals by SRB has been widely used in the bioremediation of acid mine wastewater and soil (Villegas-Plazas et al., 2019).

### Taxonomic compositions of bacterial communities in different remediation measures

**Figure 2:**
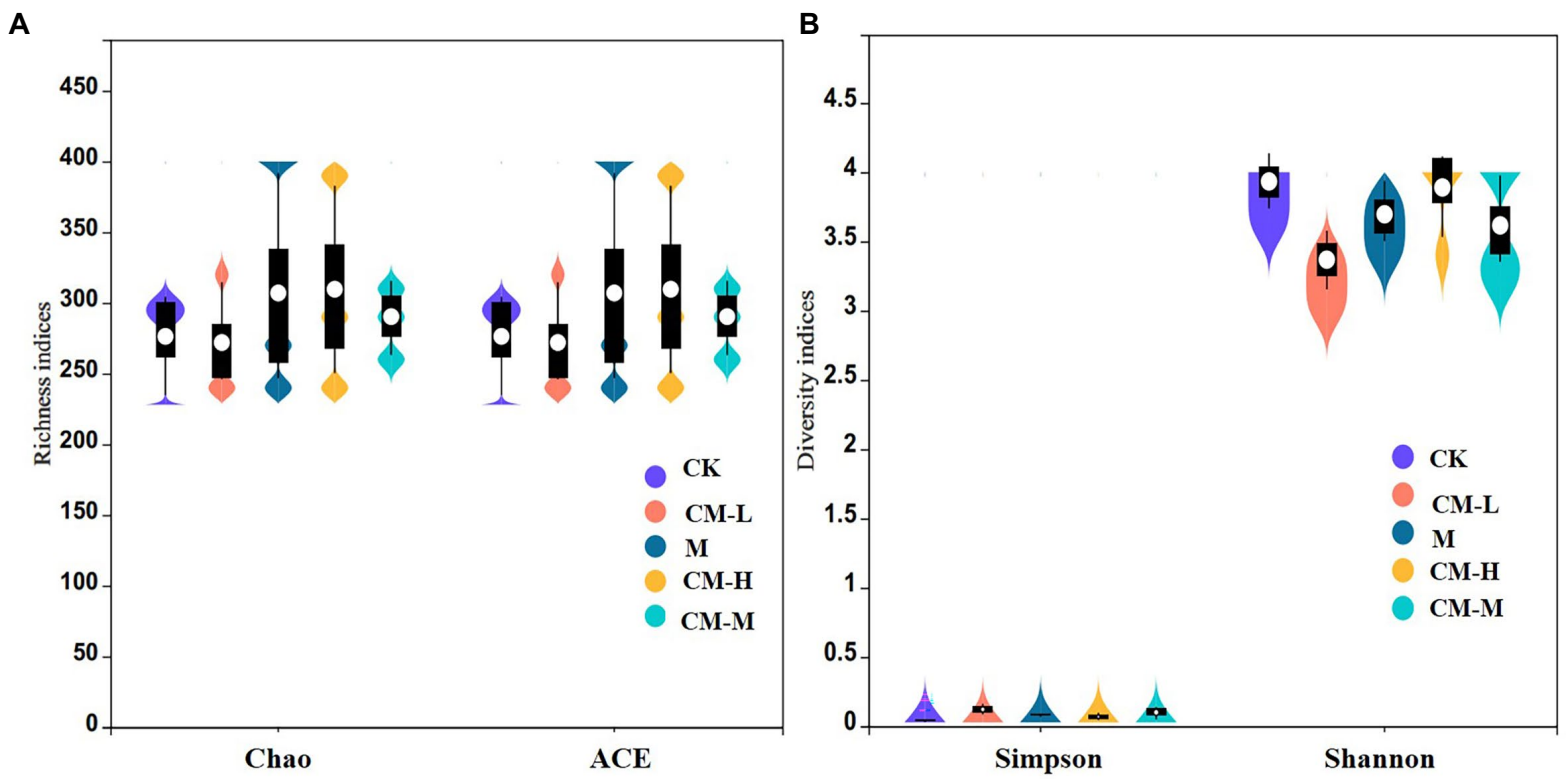
Alpha-diversity of samples collected from different remediation measures. **(A)** Richness indices (ACE and Chao) and **(B)** diversity indices (Simpson and Shannon).

Sequencing of the V4 region of the 16S rDNA yielded a total of 835,812 high-quality reads for 15 samples of five different remediation measures. The richness indices (ACE and Chao) and diversity indices (Simpson and Shannon) were compared among different remediation measures (Figure 2). In comparison with the CK, ACE and Chao indices were significantly increased, which was attributed to the inoculation of SRB increase in the number of system bacterial species (Yin et al., 2022). Meanwhile, the collaborative remediation groups of CM-L, CM-M, and CM-H presented higher richness indices compared to the M. This was explained that the improvement of the harsh environment (strongly acidic soil) can provide a better living environment for various bacterial colonization (Zhu et al., 2022). In addition, sufficient nutrients can stimulate the growth and metabolism of rare indigenous microorganisms whose abundance was not counted previously (Xu et al., 2022; Yin et al., 2022). For the variation of diversity indices in different remediation measures, there was a significant decreasing trend for the Simpson and Shannon diversity indices with the inoculation of functional microbes, with the M having the least bacterial diversity indices, followed by the CM-H, CM-L, and CM-M. These results were consistent with the previous study (Koner et al., 2022). Artificial inoculation of functional microbes was a bioaugmentation process used to regulate indigenous bacterial community structure in the soil, resulting in several bacterial species with strong competitiveness occupying ecological niches while rare microbes with weak competitiveness did not survive (Chen et al., 2022). Moreover, improvements in soil quality may indicate that bacterial species with an ability to adapt to circumstances rapidly were enriched, whereas other bacterial taxa showed the opposite trend (Zong and Fu, 2021).

**Figure 3:**
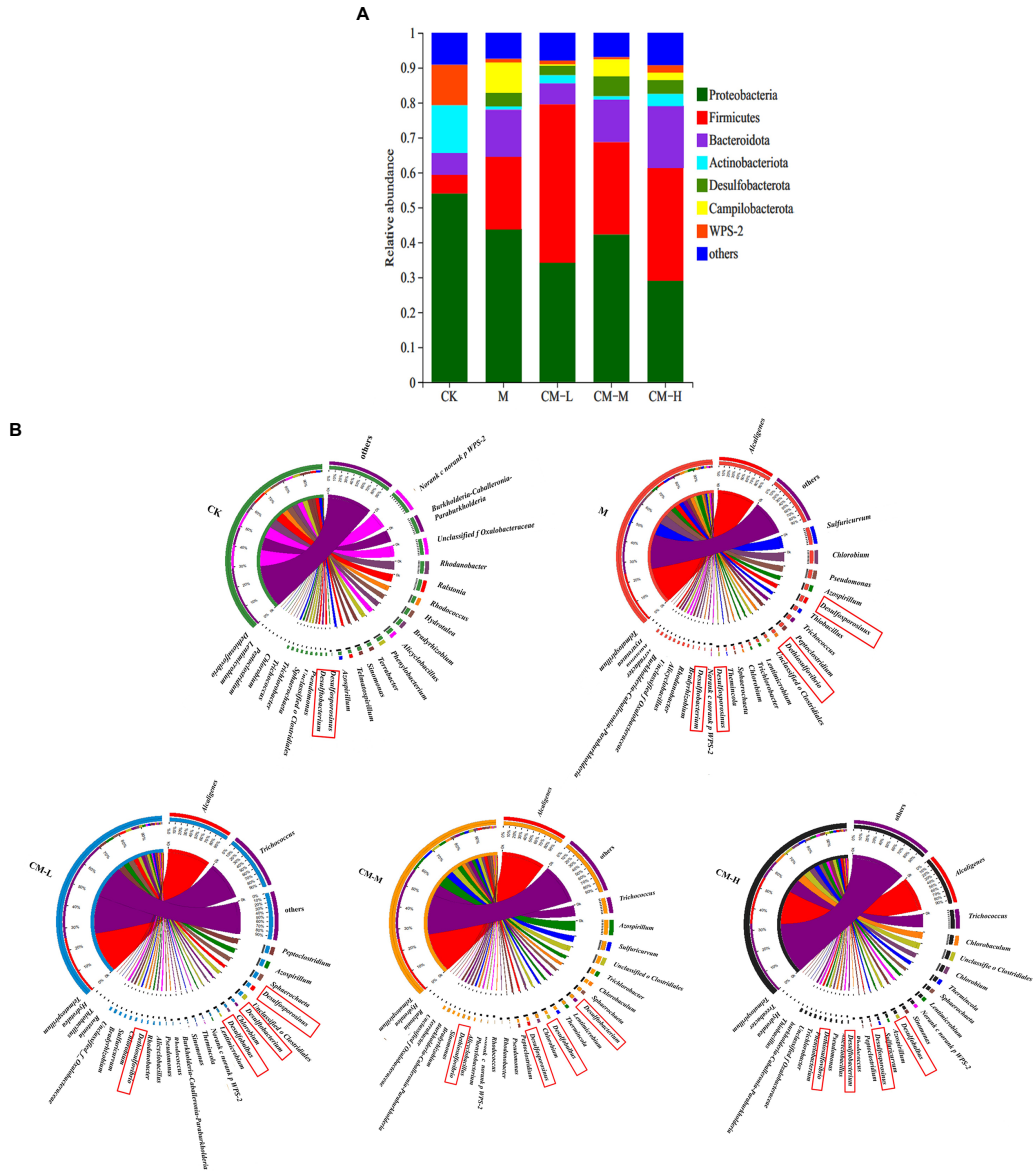
Relative abundances of bacterial community at the level of phylum **(A)** and genus **(B)** in the REM soil samples under different treatments.

Barplots and Circos graphs showed bacterial community composition and abundance at the phylum and genus levels of bacteria (Figure 3). Dominant bacterial compositions of phylum (>1% relative abundance in at least one treatment) are shown in Figure 3A, and those with less than 1% relative abundance were classified as “others.” Six major phyla were detected including Proteobacteria (29–54%), Firmicutes (5–45%), Bacteroidota (6–18%), Actinobacteriota (1–14%), *Desulfobacterota* (0.03–6%), and Campilobacterota (0.05–8%), which totally accounted for a high proportion above 90%. Chen et al. (2021) found that Proteobacteria, Firmicutes, Bacteroidota, and Actinobacteriota were the most common bacterial species found in contaminated soil (Chen et al., 2021). This was consistent with our study that Proteobacteria, Firmicutes, Bacteroidota, and Actinobacteriota were three major phyla in the CK. The difference was a high richness of Desulfobacterota and Campilobacterota presenting in the M, CM-L, CM-M, and CM-H. This may be related to Desulfobacterota and Campilobacterota belonging to the common phylum of SRB. Inoculation of SRB results in a significant increase in abundance. According to the variation in contaminant concentration, functional microbes play an important role in the remediation process of SO_4_^2−^ and heavy metals. Scholars discovered that Desulfobacterota was the key phylum for removing SO_4_^2−^ and solidified heavy metals in the acid mine drainage (Santos et al., 2022). The relative abundance of Desulfobacterota in the CM-L, CM-M, and CM-H was higher than the M, which explained that the physical and chemical properties of soil were improved rapidly by the addition of Ca(OH)_2_, and then, inoculated functional microbes were easier to survive and produce a marked effect. With the different concentrations of Ca(OH)_2_, the abundance of Desulfobacterota was presented as CM-M > CM-L > CM-H, which was attributed to Desulfobacterota’s inability to grow well in an acidic or alkaline environment. It was found by Li J. et al., (2022) that *Desulfobacterota* had a high abundance when the pH value of the system was 6–9 (Li W. et al., 2022).

To further characterize the responses of bacterial communities to different remediation measures, genus-level analysis was performed by Circos graphs to determine the differences in functional microbes (Figure 3B). In this study, the key genus including *Desulfosporosinus*, *Desulfitobacterium*, *Desulfobulbus*, and *Dethiosulfovibrio* belonged to Desulfobacterota. These microbes could convert SO_4_^2−^ into H_2_S and may be capable of absorbing heavy metals in their water-soluble state (Du et al., 2022). At the genus level, the relative abundance of *Desulfosporosinus*, *Desulfitobacterium*, *Desulfobulbus*, and *Dethiosulfovibrio* varied greatly in conjunction with different remediation measures. The bacterial community composition was similar to that of the bioremediation groups from acid mine drainage treatment but differed from the treatment of contaminated soil of heavy metals. In comparison with the CK (0.005%), the total abundance proportion of *Desulfosporosinus*, *Desulfitobacterium*, *Desulfobulbus*, and *Dethiosulfovibrio* was relatively higher and presented as the M (7.58%) > CM-M (6.13%) > CM-L (6.11%) > CM-H (3.91%). For the M, the relatively high abundance of *Desulfobacterota* may be attributed to the priority effects of bacterial colonization in the ecological theory. When the population of functional microbes was inoculated into the M, functional bacterial communities would prioritize colonization in the current environment by improving competitiveness and trophic resources. Overall, previous studies reported that predicting when and how the current community state impacts the success of newly arriving bacterial taxa was critical for the management of microbiomes to sustain ecological function (Li et al., 2021; Ma et al., 2022). The higher relative abundance of *Desulfobacterota in the* CM-M (6.13%) and CM-L (6.11%) were higher than it in the CM-H (3.91%) which due to adding of chemicals greatly changed the physicochemical properties of soil in the CM-H, and then, functional microbes need more time to resist and adapt to the environment, meanwhile, the competition of indigenous microbes was also a major obstacle affecting the colonization of *Desulfobacterota* (Geng et al., 2022). In addition, Santos et al. (2022) found that the addition of chemicals had a great influence on the abundance and diversity of bacterial communities (Santos et al., 2022). *Desulfobacterota* was the key player in the process of transforming SO_4_^2−^ into H_2_S, and then, achieving the solidification of heavy metals by H_2_S combining with heavy metal in the water-solution state to form stable sulfide in the RM soil (Lima and Ottosen, 2021). In addition, many Firmicutes were related to the solidification of heavy metals. For example, *Trichococcus* and *Bacillus* (Firmicutes) were widely used for treating the contaminated soil of heavy metals (Pb, Zn, Cu, As, and Cd) (Jiang et al., 2021). The *Alcaligenes* had a relatively high abundance in the remediation group, which had the capacity of improving the acid soil environment (Wu et al., 2021). This result was consistent with Du et al. (2022), who found that the inoculation of *Alcaligenes* in acid wastewater can decline the concentration of organic acids and increase the pH value of the systems (Du et al., 2022).

### Relationship between environmental factors and bacterial community structure

**Figure 4:**
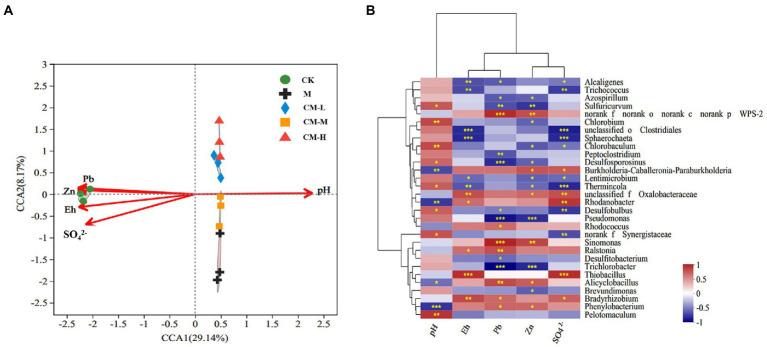
**(A)** Result from CCA to explore the relationship between soil bacterial community and soil physiochemical properties, and contaminants. **(B)** Heatmap of Spearman’s rank correlation coefficients combined with a cluster analysis between soil physiochemical properties, contaminants and the relative abundances of the bacterial genus in the top 30. Horizontal row represents soil physiochemical properties and contaminants information, vertical row represents microbial community abundance information, red represents positive correlation, blue represents negative correlation, darker color indicates higher correlation, value of *p* is the correlation test result, * in the figure indicates *p* < 0.05, and ** indicates *p* < 0.01. *** in the figure indicates *p* < 0.001.

Canonical correlation analysis (CCA) by chi-square distance was used to reflect the relationship between different environmental factors and bacterial community (on the genus level). As shown in Figure 4, environmental factors (SO_4_^2−^, Pb, Zn, pH, and Eh) were chosen for CCA analysis. Five combinational variables accounted for 37.31% of observed changes in bacterial community composition, with axis 1 accounting for 29.14% and axis 2 accounting for 8.17%. The distributions of the bacterial community under the M, CM-L, CM-M, and CM-H were negatively correlated with Eh, SO_4_^2−^, Pb, and Zn (Figure 4A). It means that the relative abundance of the bacterial community increased with the decline of SO_4_^2−^, Pb, and Zn concentrations. Based on previous studies, Huang et al. (2022) concluded that the population and activity of microbes for bioremediation were the main factors in the process of removing SO_4_^2−^ and heavy metals (Huang et al. 2022; Zhang et al., 2022). The distributions of the bacterial community in the CK were all positively correlated with all factors. This is consistent with previous results about the richness index and the concentration of SO_4_^2−^, Pb, and Zn in the CK. Due to the lack of management of the closed REM for a long time, many contaminants containing heavy metals and SO_4_^2−^ continued to dissolve as a result of the action of acid rain. This was causing a decline in the richness and diversity of the bacterial community. In addition, the bacterial community structure in all treatments, except CK, was significantly and positively correlated with the pH value, which was explained as a process of consuming acid for transforming SO_4_^2−^ by the SRB (Tang et al., 2022).

A Spearman’s correlation analysis between the bacterial community structure and the main factors was calculated (Figure 4B). According to the descending order of bacterial abundance, the majority of the top 30 genera had a positive correlation with the pH value, indicating that these microbes can contribute to the improvement of soil pH. Due to potential resistance to the harsh environment and acid consumption in self-metabolism, many bacterial species can improve their pH value and colonize into the acid-contaminated system (Zhu et al., 2022). In particular, these key bacterial species involving *Desulfosporosinus*, *Desulfitobacterium*, and *Desulfobulbus* showed a negative correction with all factors, except for the pH above result indicated that these microbes can tolerate heavy metals and high sulfate conditions. A negative correction between functional microbes and Eh value, indicating that *Desulfosporosinus*, *Desulfitobacterium*, and *Desulfobulbus* can transform oxidation (Eh > 0 mV) into reduction condition (Eh < 0 mV) by reducing SO_4_^2−^ into H_2_S. Previous studies found that functional bacterial species through the reduction of SO_4_^2−^ into H_2_S had an influence on Eh value, which was transforming oxidation into reduction potential, reaching below 0 and maintaining reduction conditions. The earlier result will contribute to the sustainable prevention of heavy metal dissolution (Minari et al., 2020; Gu et al., 2021). A significantly negative correlation between functional bacterial species and Pb and Zn was attributed to these microbes producing enough H_2_S to combine with heavy metal, causing the decline of Pb and Zn concentrations. Sinharoy and Pakshirajan (2019) found continuous accumulation of stable sulfide (PbS and ZnS) with the production of H_2_S. In addition, the negative correlation between functional bacterial species and SO_4_^2−^ was due to these microbes’ need for abundant SO_4_^2−^ for growth, proliferation, and self-metabolism (Santos et al., 2022). With the consumption of SO_4_^2−^, the population and activity of functional microbes were continuously increasing. It was achieving the goal of removing SO_4_^2−^ and solidification of heavy metals in the system.

### The differences of bacterial metabolic function in different remediation measures

**Figure 5:**
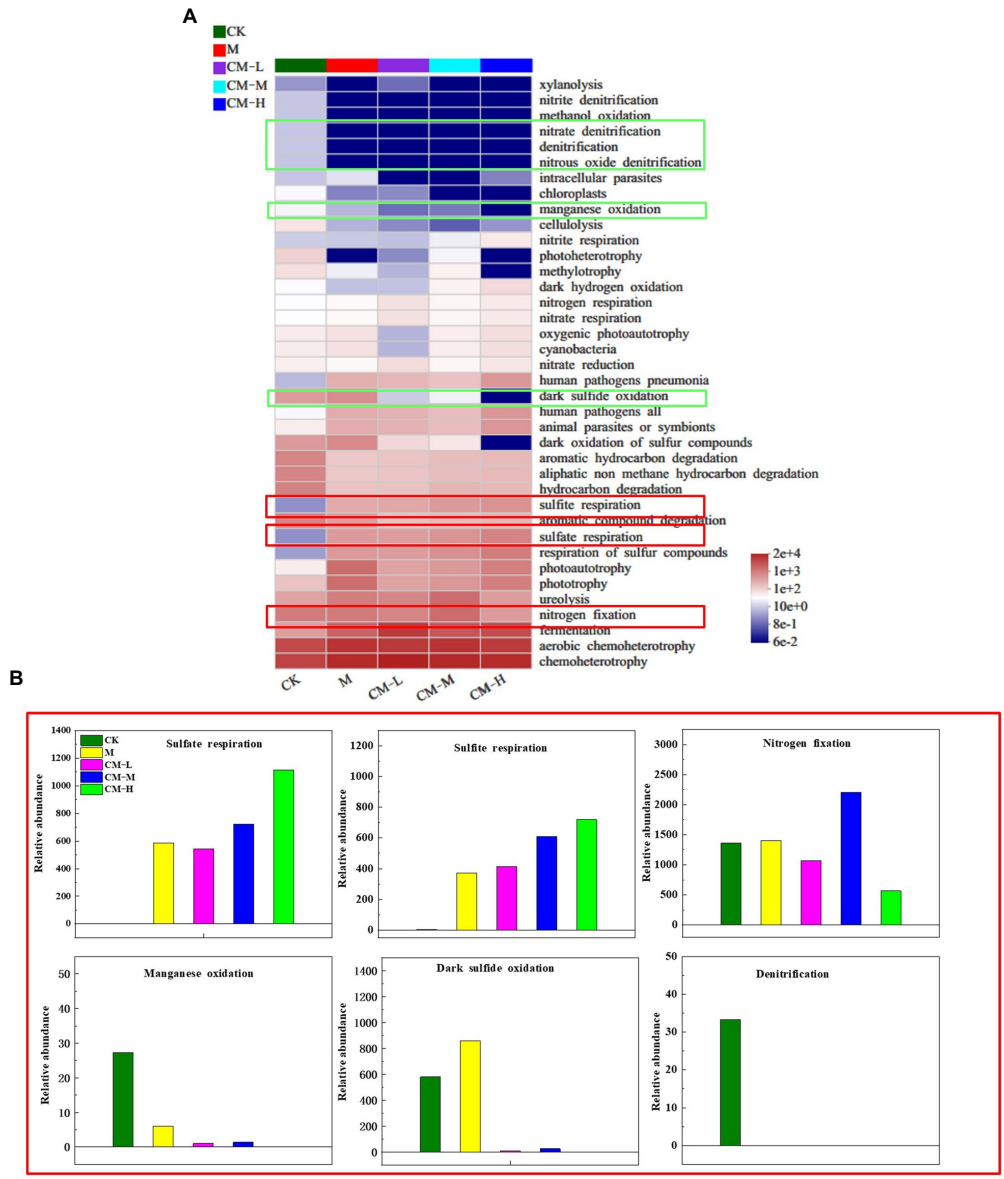
Microbial metabolism profiles in the REM soil with different treatments. **(A)** Heatmap analysis of the top 40 differential metabolites. **(B)** The relative abundance differences in different functional groups.

In order to compare the difference in functional characteristics in different remediation measures, the functional annotation of prokaryotic taxa (FAPROTAX) was used to predict prokaryotic clades to establish metabolic or micro-ecological relevant functions under different remediation measures. A total of 56 functional groups in the FAPROTAX database were identified, and the relative abundance of 34 out of 38 was differences in the dotted boxes among different remediation measures (Figure 5A). Therefore, these functional groups were defined as sensitive functional groups in different remediation measures. Of the 34 sensitive functional groups in the different remediation measures, six functional groups, including sulfate respiration, sulfite respiration, nitrogen fixation, manganese oxidation, dark hydrogen oxidation, and denitrification, were significantly different (Figure 5B). Among them, three functional groups of sulfate respiration, sulfite respiration, and nitrogen fixation were significantly increased after the inoculation of functional microbes in comparison with the CK. This result was related to the high SO_4_^2−^ concentration of the system. SO_4_^2−^ and SO_3_^2−^ often acted as electron acceptors to participate in the respiration of functional microbes with the capacity of transforming SO_4_^2−^ into H_2_S. Previous studies concluded that the increase of sulfate respiration and sulfite respiration was related to the relatively high abundance of *Desulfosporosinus*, *Desulfitobacterium*, *Desulfobulbus*, and *Dethiosulfovibrio* in the high SO_4_^2−^ system (Gao et al., 2022; Santos et al., 2022). The difference in nitrogen fixation in different remediation measures may be attributed to the stimulation of nutrients and nitrogen-fixing microorganisms (Azospirillum). After adjusting pH by Ca(OH)_2_, the addition of nutrients stimulated the growth and activity of ingenious microbes with nitrogen fixation ability in a suitable pH of the system. This was the main reason why nitrogen fixation content presented as CM-M > CM-L > CM-H. In comparison with the CK, another three groups, such as manganese oxidation, dark hydrogen oxidation, and denitrification, had a declining trend with the inoculation of functional microbes. Oxidizing microbes associated with the manganese oxidation metabolism can promote the dissolution of heavy metal (Mn), which then significantly increases in the oxidized conditions with the abundance of these microbes. In this study, the initial oxidized conditions in the M, CM-L, CM-M, and CM-H transformed gradually into the reduction conditions by the inoculation of functional microbes and caused the decline of oxidizing bacterial abundance, and that was the reason for the decrease of manganese oxidation metabolism in all treatments, except for the CK. The difference in denitrification metabolism between the CK and the others was attributed to the high abundance of denitrification bacteria (Alcaligenes, Burkholderia-Caballeronia-Paraburkholderia, Pseudomonas, and Rhodococcus) in the CK. Previous studies reported that Pseudomonas, Alcaligenes, and Rhodococcus could promote the conversion of nitrate nitrogen to nitrogen.

## Conclusion

In this study, the concentration of contaminants from the REM soil was decreased in different remediation measures. The results indicated that the CM-M had a more efficient removal effect for SO_4_^2−^, Pb, Zn, and Mn than the others, up to 94.6, 88.3, 98.7, and 91%, respectively. The difference in bacterial community structure in different remediation measures was detected. Compared with the CK, *Desulfobacterota* with the ability to transform SO_4_^2−^ into S^2−^ increased significantly. The correlation between environmental factors and bacterial community structure is presented as follows: SO_4_^2−^ > Pb > pH > Zn > Eh. Among them, Eh, SO_4_^2−^, Pb, and Zn had a negative correlation with distributions of bacterial communities in the M, CM-L, CM-M, and CM-H. Functional prediction analysis showed significant differences in the five treatments. The functional groups’ abundance including sulfate respiration, sulfite respiration, and nitrogen fixation significantly increased in all treatments, except for the CK, while the manganese oxidation, dark hydrogen oxidation, and denitrification decreased. The study provides an effective method for the removal of contaminants from the REM soil and establishes the theoretical foundation for harmlessness and reclamation of the REM.

## Data availability statement

The original contributions presented in the study are included in the article. Because part of this batch of original data involves other research topics and paper publication, further inquiries can be directed to the corresponding author.

## Author contributions

MZ provided the idea of this work. XY and BG performed the experiments, collected the samples, detected, analyzed the data, and involved in experimental design. XY prepared the figures and wrote the manuscript. XZ detected physiochemical properties. BG collected the samples. JW revised the manuscript. All authors contributed to the article and approved the submitted version.

## Funding

This project was financially supported by the National Natural Science Foundation of China (grant number 51974279), the National Key Research & Development Program of China (grant numbers 2018YFC18018 and 2018YFC18027), and GRINM Youth Fund (grants number 12208), which are greatly appreciated.

## Conflict of interest

XY, BG, JW, XZ, and MZ were employed by GRINM Resources and Environment Tech. Co., Ltd. and GRINM Group Co., Ltd. XZ was employed by GRIMAT Engineering Institute Co., Ltd.

## Publisher’s note

All claims expressed in this article are solely those of the authors and do not necessarily represent those of their affiliated organizations, or those of the publisher, the editors and the reviewers. Any product that may be evaluated in this article, or claim that may be made by its manufacturer, is not guaranteed or endorsed by the publisher.

## Footnotes

^1^ https://cloud.majorbio.com/page/tools/
